# Anxiety and attitudes toward help-seeking: the mediating roles of mental health literacy and stigma in a Romanian sample

**DOI:** 10.3389/fpsyg.2026.1812675

**Published:** 2026-08-19

**Authors:** Alex Armand Hohn, Laurentiu Maricutoiu

**Affiliations:** Faculty of Psychology and Educational Sciences, West University of Timisoara, Timișoara, Romania

**Keywords:** anxiety, help-seeking attitudes, help-seeking propensity, mediation analysis, mental health literacy, mental health stigma, self-efficacy

## Abstract

This study investigates the relationship between anxiety and professional help-seeking attitudes, examining the mediating roles of mental health literacy (MHL) and stigma within a Romanian context. Grounded in the Theory of Planned Behavior and the ABC model of attitudes, the research explores how cognitive (MHL) and affective (stigma) components predict help-seeking propensity, psychological openness, and indifference to stigma. Data were collected from 351 Romanian participants. Correlation analyses revealed that anxiety is positively associated with psychological openness and mental health knowledge, and it is negatively linked to help-seeking skills and self-help strategies. Mediation models suggested that anxiety predicts greater psychological openness but lower help-seeking propensity, indicating that emotional distress may increase self-awareness without necessarily fostering behavioral intention. MHL demonstrated dual, opposing effects: while increased knowledge enhanced help-seeking propensity, lower perceived help-seeking skills significantly hindered it. These findings suggest that knowledge alone is insufficient to facilitate action unless accompanied by self-efficacy and skill-building. The results underscore that cognitive and affective pathways operate differently across help-seeking dimensions. This research provides culturally relevant evidence for a Romanian convenience sample, highlighting the need for interventions that go beyond traditional psychoeducation by integrating skill-building and strategies to mitigate internalized and anticipated stigma. Despite limitations regarding its cross-sectional design and convenience sampling, the study offers critical insights into the complex mechanisms shaping help-seeking intentions.

## Introduction

Recent studies concluded that most people dealing with a mental disorder do not get professional help. A systematic review of literature regarding help-seeking concluded that 54 to 74% of the people who suffer from a mental disorder are not receiving specialized treatment, across all Europe and America (Clement et al., 2015). This phenomenon is called the “unmet need” in treatment (Andrews and Henderson, 2000; Wang et al., 2005), and might be relevant for disorders that have a high prevalence and also can be associated with significant disability.

Anxiety disorders (AD) have the highest prevalence of all mental health disorders (with an estimated prevalence of ~4%) and are responsible for a high degree of disability (GBD 2019 Diseases and Injuries Collaborators, 2020). Clinical evidence indicates that individuals suffering from ADs report significant functional impairment (Mack et al., 2015) and face an alarming average delay of 6 years between symptom onset and first service use (Mack et al., 2014). In the case of ADs, this pervasive “unmet need” cannot be attributed to a scarcity of validated treatments, given the abundance of successful pharmacological and evidence-based psychological interventions, such as cognitive-behavioral therapy (Baldwin et al., 2014; Carpenter et al., 2018). Nevertheless, professional help-seeking rates for anxiety have failed to increase significantly over the last three decades (Clement et al., 2015; Heinig et al., 2021; Johnson and Coles, 2013).

Crucially, these long delays suggest that by the time anxiety reaches clinical thresholds, it triggers deeply entrenched avoidance mechanisms that actively paralyze help-seeking. This underscores a critical requirement to shift scientific attention toward the pre-clinical window of opportunity. Rather than investigating populations already locked in clinical avoidance, it is vital to examine how sub-clinical, transient anxiety symptoms influence help-seeking determinants within the general population. Understanding these early, non-clinical stages of distress provides a strategic entry point for early preventive interventions, capturing individuals before their transient discomfort chronicizes into long-term avoidance.

A recent systematic review reported that there are numerous barriers toward help seeking: (i) mental health literacy (MHL), (ii) lack of money, and (iii) stigma (Hohn and Maricutoiu, 2024). However, the review relied on data predominantly collected on USA, Canadian and Australian populations, due to the paucity of studies conducted on other cultures. The lack of studies can restrict the generalizability of their findings to a more heterogeneous and diverse population (Hohn and Maricutoiu, 2024).

The present study aimed to provide evidence from cultures that have been underrepresented in previous systematic reviews. While cross-cultural variability in actual help-seeking rates is well-documented—often due to differing healthcare infrastructures, socio-economic factors, or societal norms—the core cognitive and affective mechanisms driving these behavioral intentions may share universal pathways. In the present research, we do not specifically expect these underlying psychological mechanisms to operate fundamentally differently in the Romanian context compared to Western populations. Rather, confirming this structural similarity is essential. By testing these relationships in an underrepresented Eastern European population, this research aimed to validate the cross-cultural robustness and generalizability of the theoretical models utilized, demonstrating their applicability beyond predominantly Western, Educated, Industrialized, Rich, and Democratic (WEIRD) societies. Therefore, the current study examined the influence of mental health literacy and stigma on attitudes toward help-seeking behaviours.

As shown in numerous studies the cognitive component of The ABC model such as mental health literacy plays an important role in shaping attitudes toward help seeking (Ostrom, 1969; Breckler, 1984; Al-Shannaq and Aldalaykeh, 2023; Calear et al., 2021; Jung et al., 2017). Also, stigma has an emotional component (e.g., anxiety, social fear, embarrassment, etc.) and can influence attitudes toward help-seeking by shaping the affective component regarding one’s attitude in relation to help-seeking (Han et al., 2017; Chen, 2018; Luo et al., 2018). The ABC model showed that attitudes are influenced by affect, cognition and behavior of one’s self toward an attitude object (Kaiser and Wilson, 2019). In other words, to influence the attitudes toward help-seeking, one must influence the affective, cognitive, or behavioral component of one’s self, regarding help-seeking.

The significance of the present study lies in its focus on the persistent ‘treatment gap’ within the Eastern European mental health landscape, where rates of professional help-seeking in Romania remain disproportionately low compared to Western standards (Vlădescu et al., 2016; Winkler et al., 2017). This discrepancy suggests that clinical distress alone is insufficient to trigger service engagement, pointing toward the existence of deep-seated cognitive and attitudinal barriers such as pervasive public and self-stigma, which are well-documented barriers to mental health care in both Romania and the broader post-communist Eastern European context (Hohn and Maricutoiu, 2024; Winkler et al., 2017). To address this, the current research adopts a multidimensional theoretical framework, conceptualizing the relationship between anxiety and help-seeking not as a simple, uniform vector, but as a complex process operating through competing psychological pathways. Specifically, we propose that anxiety can simultaneously facilitate and inhibit help-seeking attitudes depending on how it interacts with other psychological factors: it may drive the need for information (thereby increasing mental health literacy and help-seeking propensity), while concurrently activating self-stigma or avoidance (thereby hindering propensity). By examining the interplay between mental health literacy and various dimensions of stigma, this research moves beyond descriptive analysis to identify the specific and sometimes opposing, mechanisms that either facilitate or hinder the transition from experiencing symptoms to seeking care. Understanding these competing pathways is crucial for developing targeted, culturally sensitive interventions that can transform psychological openness into actual behavioral intention, within a Romanian convenience sample,ultimately reducing the societal and individual burden of untreated anxiety.

### Attitudes toward help-seeking behaviours

Attitudes toward seeking mental health services reflect individuals’ evaluations, beliefs, and behavioral tendencies regarding the use of professional psychological support (Ajzen and Fishbein, 2000). In a broader sense, attitudes represent an individual’s evaluative judgment of a given object or behavior and are understood as a multidimensional construct encompassing affective, cognitive, and behavioral components (Rosenberg et al., 1960). This tripartite structure highlights the importance of examining not only what individuals think about a behavior, but also how they feel about it and how likely they are to act upon those evaluations (Perry et al., 2022).

A general framework for understanding how such attitudes are translated into behavior is provided by the Theory of Planned Behavior (TPB) (Ajzen, 1985; Ajzen, 2020). According to TPB (Ajzen, 1985), behavior is influenced by three core determinants: attitudes toward the behavior; (i.e., positive or negative evaluations of performing the behavior); subjective norms (i.e., perceived social pressure to engage or not engage in the behavior); and perceived behavioral control (i.e., individuals’ perceptions of how easy or difficult it is to perform the behavior). Together, these components shape behavioral intentions, which constitute the most proximal predictor of actual behavior.

When applied to the domain of mental health, TPB provides a useful framework for understanding professional help-seeking. In this context, individuals’ attitudes toward help-seeking capture their evaluations of seeking psychological support, subjective norms reflect perceived approval or disapproval from significant others, and perceived behavioral control refers to beliefs about one’s ability to access and engage with mental health services. These factors jointly influence intentions to seek help, which in turn increase the likelihood of actual help-seeking behavior (Ajzen, 2020).

Consistent with this framework, Mackenzie et al. (2004) operationalized attitudes toward professional psychological help-seeking across three distinct dimensions: help-seeking propensity (willingness and perceived capability to seek professional assistance), psychological openness (acknowledgment of psychological difficulties), and indifference to stigma (the degree to which social judgment is disregarded). To capture the complex interplay behind these dimensions, the current study explicitly integrates the ABC model of attitudes and the Theory of Planned Behavior (TPB) into a unified conceptual matrix. Specifically, we map mental health knowledge as the cognitive component, stigma-related processes as the affective component reflecting the social pressures of subjective norms, and practical help-seeking skills as the behavioral component directly underpinning perceived behavioral control (PBC).

This integrated framing is essential to explain how emotional distress shapes help-seeking, particularly when symptoms are non-clinical. While severe, chronic anxiety triggers behavioral avoidance, the acute discomfort of transient anxiety symptoms often acts as a cognitive and affective catalyst. It heightens an individual’s awareness of their psychological state (enhancing the cognitive/affective alignment toward psychological openness) and creates an internal drive for symptom relief. However, within the TPB framework, an increased need or a favorable attitude is insufficient to drive action if the behavioral component—perceived behavioral control—is compromised by a lack of practical navigation skills. Models of help-seeking emphasize that the appraisal of distress is merely the initial step (Rickwood et al., 2007); therefore, mapping these variables onto the ABC and TPB frameworks allows us to explore whether transient distress acts as a uniform facilitator or if it introduces a structural misalignment between attitudinal openness and behavioral control.

Based on these theoretical considerations, the following hypothesis was formulated:

*H1*: Anxiety levels are positively associated with attitudes toward seeking professional psychological help.

### Mental health literacy

Mental health literacy (MHL) was first defined in 1997 as ‘knowledge and beliefs about mental disorders which aid their recognition, management or prevention’ (Jorm et al., 1997). The concept of MHL has several components, as follows: (a) the ability of recognizing different mental health disorders or types of psychological distress, (b) knowledge and beliefs about mental health risk factors and causes of mental health illness, (c) knowledge and beliefs about self-help, (d) knowledge and beliefs about professional help (e) attitudes toward mental health disorder recognition and help-seeking, and (f) knowledge about seeking mental health information (Jorm, 2000).

A number of studies conducted on MHL showed that the failure of individuals to seek help from a mental health professional is strongly correlated with one’s inability to recognize mental health symptoms and with erroneous beliefs about their symptom outcomes or the ‘right’ course of action (Coles and Coleman, 2010). A study conducted in Australia showed that the ‘lack of knowledge’ was the main reason why 60% of individuals dealing with a mental health disorder delayed the help seeking process (Thompson et al., 2004). Also, low levels of MHL correlated with high levels of depression and anxiety (Zhong et al., 2024). A systematic review of 22 studies concluded that low MHL is a significant barrier toward developing positive attitudes toward help seeking behaviours (Gulliver et al., 2010). As shown in a recent study on the Chinese population, there is a positive association between MHL and the attitudes toward seeking professional help (Yang et al., 2023).

Previous research indicates that anxiety is associated with a greater preference for novel or information-rich options, particularly in situations involving ambiguity or perceived risk (Hartley and Phelps, 2012). This suggests that individuals experiencing anxiety may demonstrate an increased need for information. As a result, such exploratory behavior may generalize to the investigation of professional help-seeking avenues as a means of gaining information and restoring a sense of control over anxiety-related distress (Witte et al., 2024). Therefore, we formulate the second and third hypothesis of the present study:

*H2*: Self-reported anxiety level is correlated with mental health literacy.

*H3*: Mental health literacy is a mediator between feeling anxiety and attitudes toward professional help-seeking.

### Stigma

Stigma has its origin in ancient Greek and Roman societies and originally referred to tattoos and marks burnt or cut into the skin of slaves or people having criminal records, as a testament for their social marginal status (Link and Phelan, 2001). Current conceptualizations of stigma include three distinct types of manifestations: (a) public stigma – refers to a social process in which attitudes and reactions regarding an individual or a group of individuals are shared or perceived to be shared by the general community (Clement et al., 2015; Curcio and Corboy, 2020), (b) personal stigma – refers to one’s one beliefs and attitudes regarding an individual or a group of individuals (Aromaa et al., 2011; Curcio and Corboy, 2020), and (c) self-stigma – refers to labeling oneself by internalizing of stigmatized attitudes, beliefs and stereotypes (Corrigan and Watson, 2002; Corrigan and Shapiro, 2010).

Stigma regarding attitudes toward professional help-seeking refers to the negative labels put on individuals or groups of individuals that are seeking professional help for mental health problems (Corrigan, 2004). This phenomenon is commonly conceptualized as manifesting in two primary forms: public stigma and self-stigma (Keum et al., 2018). The well-being of an individual is closely related to mental health illness stigma. Individuals can feel stigmatized in different domain of their lives: (a) employment, where they can experience direct discrimination from coworkers and employers or indirect discrimination such as policy negligence (Stuart, 2006); (b) interactions with law enforcement where the most common sources of discrimination were mental disability, race, sexual orientation and other disabilities (Corrigan et al., 2003) and (c) medical care which consists of negative attitudes amidst medical professionals regarding their prognosis (Thornicroft et al., 2007). Experiencing stigma is commonly associated with increased symptom severity, low levels of help-seeking or low adherence of treatment (Boyd et al., 2014; Fox et al., 2018).

A study by Ociskova et al. (2015), concluded that the overall level of self-stigma was positively associated with more intense symptoms of anxiety and depression. This finding was further corroborated by a subsequent article, which revealed that higher levels of anxiety disorder stigma correlated with more severe anxiety disorder symptoms, underscoring a robust direct link (Ociskova et al., 2018). Consequently, individuals with anxiety disorders appear particularly susceptible to self-stigma, a critical factor that can lead to significant delays in seeking or initiating treatment.

We formulate the fourth and the fifth hypothesis of the study:

*H4*: The intensity of anxiety felt by one individual is correlated with greater stigma.

*H5*: Stigma is a mediator between feeling anxiety and attitudes toward professional help seeking.

### The present study

**Figure 1:**
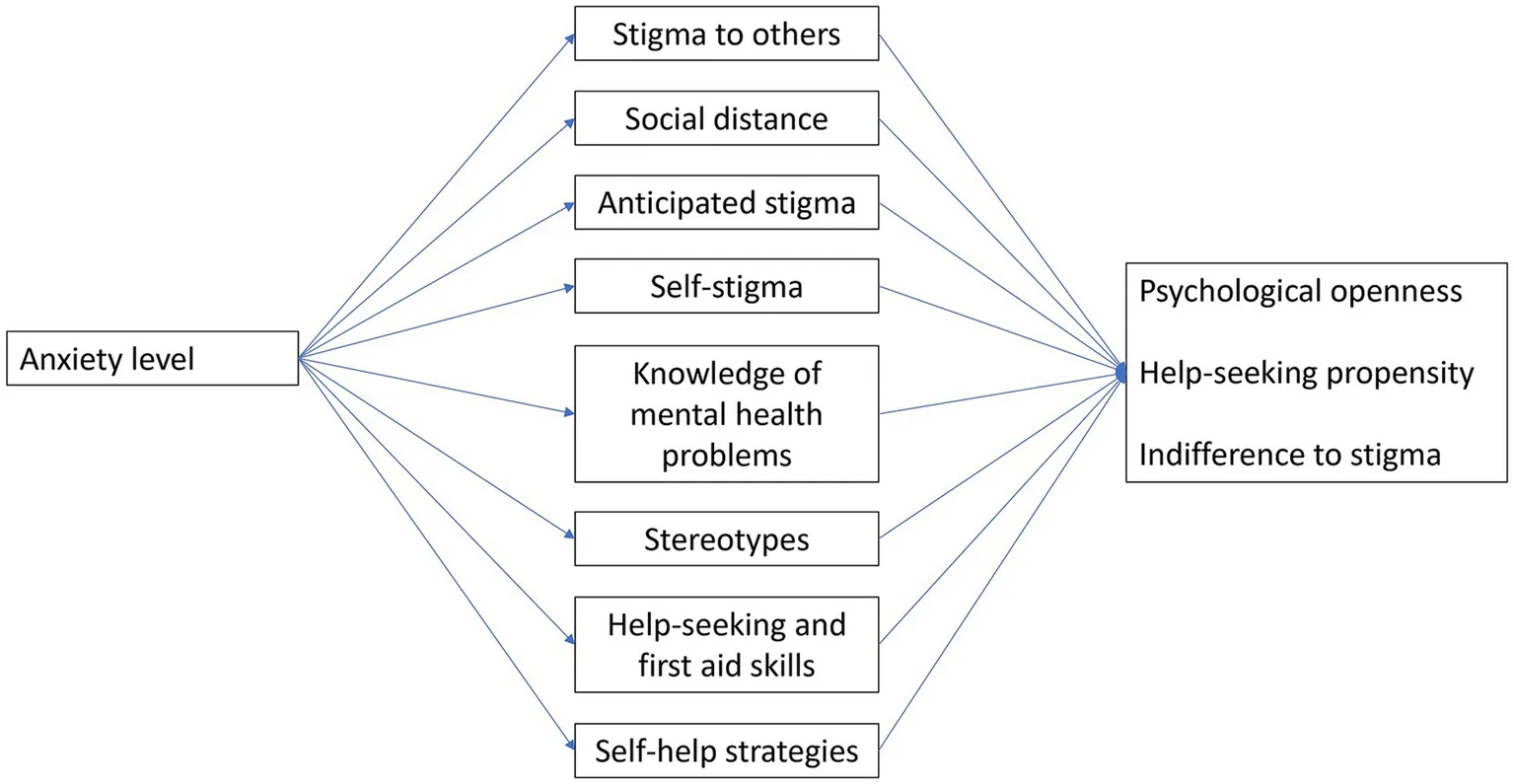
Conceptual diagram illustrating the mediating roles of mental health literacy and stigma in the relationship between anxiety and attitudes toward professional help-seeking.

We collected data from Romanian residents to investigate a model that explains the relationships between the levels of anxiety felt by a person and professional psychological help-seeking attitudes (psychological openness, help seeking propensity and indifference to stigma) through the mediating effects of and psychological help-seeking stigma (a) public stigma and (b) self-stigma and mental health literacy (a) the ability of recognizing different mental health disorders or types of psychological distress, (b) knowledge and beliefs about mental health risk factors and causes of mental health illness, (c) knowledge and beliefs about self-help, (d) knowledge and beliefs about professional help (e) attitudes toward mental health disorder recognition and help-seeking, and (f) knowledge about seeking mental health information), see Figure 1.

## Materials and methods

### Participants

We conducted a cross-sectional study on a Romanian convenience sample. After receiving informed consent (which for adolescent participants age 14 to 17 was explicitly obtained from their parents or legal guardians), we asked participants to complete an online questionnaire. The sample included 351 participants (263 women, 85 men, 2 non-binary and 1 questioning). The sample was distributed as follows, 282 (80.3%) of the responders from urban areas and 69 (19.7%) of the responders from rural areas. The distribution on age groups can be seen in Table 1.

**Table 1:** The distribution on age groups of the respondents.

Age	Count	Proportion
14–17	2	0.6%
18–25	39	11.1%
26–30	30	8%
31–40	102	29.1%
41–50	113	32.2%
51–65	59	16.8%
>65	6	1.7%

The demographic sample characteristic is shown in Table 2. In the present research 60 participants (17.1%) are working in mental health domain (psychiatrists, psychologists, nurses, etc.), 30 participants (8.5%) have been diagnosed with a mental health disorder by a mental health specialist, 32 participants (9.1%) have undergone psychotherapy sessions and 18 participants (5,1%) have reported that they were hospitalized or have taken a treatment following a mental health diagnostic.

**Table 2:** Descriptive of socio-demographic (*N* = 351).

Variable	Category	Count
Gender	Female	263 (74.9%)
	Male	85 (24.2%)
	Non-Binary	2 (0.6%)
	Questioning	1 (0.3%)
Origin	Rural	69 (19.7%)
	Urban	282 (80.3%)
Do you work in the field of mental health (psychiatrist, psychologist, medical assistant, etc.)?	Yes	60 (17.1%)
	No	291 (82.9%)
Have you been diagnosed with a mental disorder by a mental health specialist?	Yes	30 (8.5%)
	No	321 (91.5%)
Have you been hospitalized or received medication treatment following the diagnosis of a mental disorder?	Yes	18 (5.1%)
	No	333 (94.9%)
Have you participated in psychotherapy sessions following the diagnosis of a mental disorder?	Yes	32 (9.1%)
	No	318 (90.9%)

H_a_ is proportion ≠ 0.5.

### Measures

Anxiety levels were assessed using the Romanian adaptation of the Depression Anxiety and Stress Scales (DASS-21; Lovibond and Lovibond, 1995), which has been validated in Romanian samples (Perte and Albu, 2011). The DASS-21 consists of 21 items measuring three dimensions: depression, anxiety, and stress, with each subscale comprising seven items. Participants rated the frequency of each symptom on a 4-point Likert scale ranging from 0 (*did not apply to me at all*) to 3 (*applied to me very much or most of the time*). In the present study, the anxiety subscale demonstrated excellent internal consistency (Cronbach’s *α* = 0.934).

Attitudes toward help-seeking behaviors, was assessed using the Inventory to Attitudes Toward Seeking Mental Health Services (IASMHS) (Mackenzie et al., 2004). The scale is an adapted version of the Orientations to seek professional help scale (Fischer and Turner, 1970). It consists of 24 items distributed on 3 dimensions: psychological openness, help seeking propensity and indifference to stigma. Items are ranked on a Likert scale from 0 to 4, where 0 means disagreement and 4 means agreement. A higher score reflects positive attitudes toward professional help-seeking. The reliability analysis of the dimensions showed a Cronbach’s α equal to 0.680 for psychological openness, 0.782 for help-seeking propensity and 0.813 for indifference to stigma.

Mental health literacy questionnaire-short version for adults (MHLq-SVa) (Campos et al., 2022) was used to assess MHL. MHLq-SVa contains 16 items organized in four dimensions: (i) knowledge of mental health problems, (ii) erroneous beliefs/stereotypes, (iii) help-seeking and first aid skills, (iv) self-help strategies. Participants are asked to choose the option that indicates how much they agree or disagree, using a five-point Likert scale (ranging from 1 = Strongly Disagree to 5 = Strongly Agree). The internal consistency of the dimensions of the scale showed a Cronbach’s *α* of 0.731 for knowledge of mental health, 0.671 for stereotypes, 0.774 for help-seeking and first aid skills and 0.659 for self-help strategies.

For the measurement of stigma, we used The stigma and self-stigma scales for attitudes to mental health problems (SASS) (Docksey et al., 2022). SASS contains 42 items across six domains: (i) stigma to others, (ii) social distance, (iii) anticipated stigma, (iv) self-stigma, (v) avoidant coping, and (vi) (lack of) help- seeking. Each domain has 6 questions. The responses to the questions are a 5-point Likert scale ranging from strongly disagree to strongly agree scored from 0 to 4. Some terms were reverse scored to prevent response bias. Each scale had a possible range of 0–24 with higher scores representing greater stigma or problems. We removed from the scale the help-seeking domain, as we have a dedicated questionnaire for help seeking behaviors and also the avoidant coping domain. In the validation of the SASS there was found a good internal consistency for the questionnaire, except for the avoidant coping (α Cronbach = 0.47, *ω* = 0.47) (Docksey et al., 2022). Cronbach’s α is equal to 0.707 for stigma to others, 0.654 for social distance, 0.840 for anticipated stigma and 0.842 for self-stigma.

### Statistical analyses

Statistical analyses were conducted using Jamovi (version 2.3.28) and RStudio (version 4.4.2). Analyses were performed in several steps. First, descriptive statistics and internal consistency indices (Cronbach’s *α*) were computed for all study measures to assess the reliability of the instruments. Second, confirmatory factor analyses (CFA) were conducted in RStudio using the lavaan package for the questionnaires that had not been previously validated on the Romanian population, in order to evaluate their factorial structure and measurement adequacy. Depending on the multivariate normality of the data, either Maximum Likelihood (ML) or Robust Maximum Likelihood (MLR) estimators were employed. Model fit was evaluated using multiple goodness-of-fit indices: the Chi-square statistic, the Comparative Fit Index (CFI), the Tucker-Lewis Index (TLI), the Root Mean Square Error of Approximation (RMSEA), and the Standardized Root Mean Square Residual (SRMR), based on standard psychometric recommendations (Hu and Bentler, 1999). Third, bivariate correlation analyses were performed to examine associations between anxiety, help-seeking attitudes, mental health literacy, and stigma-related variables. Finally, mediation analyses were carried out using Jamovi to test the hypothesized indirect effects of mental health literacy and stigma dimensions on the relationship between anxiety and attitudes toward professional help-seeking. Indirect effects were computed using the multivariate Delta method, following modern mediation framework recommendations that evaluate the significance of specific indirect pathways directly (Zhao et al., 2010). To address potential concerns regarding the sample’s capacity to detect small indirect effects, a post-hoc sensitivity analysis was conducted. Given our sample size of N = 315 (with α = 0.05 and the target power of 80%), the analysis indicated that the study possesses sufficient sensitivity to detect a minimum effect size of 0.022, confirming that the available sample was adequately powered for the proposed mediation models. All analyses were conducted using standardized estimates, and statistical significance was evaluated using conventional criteria (*p* < 0.05).

## Results

### Measurement Model

For IASMHS, a Confirmatory Factor Analysis (CFA) was conducted using the robust Maximum Likelihood (MLR) estimator in R. To achieve an acceptable model fit and account for semantic redundancies among items within the same subscale, five error covariances were allowed to correlate. The robust fit indices indicated an acceptable-to-good fit to the empirical data: Satorra-Bentler scaled Chi-square(244) = 419.127, *p* < 0.001 [with standard Chi-square(244) = 472.042, *p* < 0.001]; Robust CFI = 0.910 (Scaled CFI = 0.903); Robust TLI = 0.899 (Scaled TLI = 0.891); Robust RMSEA = 0.048, 90% CI [0.040, 0.056] [Scaled RMSEA = 0.045, 90% CI (0.038, 0.052)]; and SRMR = 0.056. Although the robust TLI was marginally below the strict 0.90 threshold (TLI = 0.899), when evaluated in conjunction with the excellent RMSEA and SRMR values (both well below the 0.06 cutoff), the overall measurement model demonstrated adequate construct validity on the Romanian sample.

For the SASS, a CFA was conducted using the robust Maximum Likelihood (MLR) estimator in R. To achieve an acceptable model fit and account for semantic redundancies among items within the same subscale, eight error covariances were allowed to correlate. The robust fit statistics revealed a highly acceptable absolute fit, with incremental fit indices being exceptionally close to the standard 0.90 threshold: Satorra-Bentler scaled Chi-square(215) = 473.955, *p* < 0.001 [with standard Chi-square(215) = 569.631, *p* < 0.001]; Robust CFI = 0.897 (Scaled CFI = 0.890); Robust TLI = 0.879 (Scaled TLI = 0.870); Robust RMSEA = 0.064, 90% CI [0.056, 0.072] [Scaled RMSEA = 0.059, 90% CI (0.052, 0.065)]; and SRMR = 0.068. In structural equation modeling, models with a large number of indicators per factor can yield slightly lower incremental fit indices (CFI and TLI) while maintaining excellent absolute fit indices (RMSEA and SRMR). Given that both the Robust RMSEA and SRMR are well below the conservative 0.08 cutoff, the modified SASS structure was deemed to possess satisfactory construct validity on the Romanian sample.

For the MHLq-SVa. a CFA was conducted using the standard Maximum Likelihood (ML) estimator in R, as the data assumptions for normality were met. The fit indices indicated an overall excellent fit to the empirical data: Chi-square(84) = 154.850, *p* < 0.001; CFI = 0.943; TLI = 0.929; RMSEA = 0.049, 90% CI [0.037, 0.061]; and SRMR = 0.052. All fit statistics comfortably surpassed the recommended thresholds (CFI and TLI > = 0.90; RMSEA and SRMR <= 0.08), demonstrating that the original four-factor structure of the MHLq-SVa possesses robust construct validity on the studied Romanian sample without requiring any post-hoc model modifications or error covariances.

### Preliminary and correlation analysis

As shown in Table 3, the bivariate correlation analysis revealed that anxiety levels were positively associated with higher levels of psychological openness [*r*(349) = 0.107, *p* = 0.045], anticipated [*r*(349) = 0.225, *p* < 0.001] and self-stigma [*r*(349) = 0.108, *p* = 0.043], as well as increased mental health knowledge [*r*(349) = 0.134, *p* = 0.011]. However, it was inversely related to help-seeking abilities [*r*(349) = −0.108, *p* = 0.043], the use of self-help strategies [*r*(349) = −0.112, *p* = 0.035] and help-seeking propensity [*r*(349) = −0.120, *p* = 0.024]. Individuals with greater psychological openness tended to report stronger endorsement of various stigma-related attitudes such as stigma to others [*r*(349) = 0.418, *p* < 0.001], while showing lower levels of help-seeking behaviours like mental health knowledge [*r*(349) = −0.226, *p* < 0.001], and social distancing [*r*(349) = −0.357, *p* < 0.001]. Overall, these findings provide only partial support for Hypothesis 1, indicating that anxiety is positively associated with certain attitudinal components of help-seeking (e.g., psychological openness), but is significantly and negatively associated with help-seeking propensity and other indicators reflecting actual help-seeking readiness or skills.

**Table 3:** Correlation analysis between variables.

Variable	1	2	3	4	5	6	7	8	9	10	11	12
1. Anxiety	—											
2. Psychological openness	0.107∗	—										
3. Help-seeking propensity	−0.120∗	−0.490∗∗∗	—									
4. Indifference to stigma	0.047	0.505∗∗∗	−0.419∗∗∗	—								
5. Stigma to others	−0.052	0.418∗∗∗	−0.226∗∗∗	0.423∗∗∗	—							
6. Social distance	0.063	−0.357∗∗∗	0.358∗∗∗	−0.448∗∗∗	−0.407∗∗∗	—						
7. Anticipated stigma	0.225∗∗∗	0.271∗∗∗	−0.168∗∗	0.428∗∗∗	0.329∗∗∗	−0.231∗∗∗	—					
8. Self-stigma	0.108∗	0.353∗∗∗	−0.188∗∗∗	0.483∗∗∗	0.474∗∗∗	−0.331∗∗∗	0.824∗∗∗	—				
9. Knowledge of mental health problems	0.134∗	−0.226∗∗∗	0.309∗∗∗	−0.169∗∗	−0.216∗∗∗	0.332∗∗∗	0.012	0.005	—			
10. Stereotypes	−0.043	0.329∗∗∗	−0.222∗∗∗	0.297∗∗∗	0.319∗∗∗	−0.171∗∗	−0.050	0.013	−0.360∗∗∗	—		
11. Help-seeking and first aid skills	−0.108∗	−0.317∗∗∗	0.619∗∗∗	−0.228∗∗∗	−0.063	0.213∗∗∗	−0.012	−0.013	0.173∗∗	−0.138∗∗	—	
12. Self-help strategies	−0.112∗	−0.094	0.277∗∗∗	−0.194∗∗∗	0.042	0.133∗	−0.033	0.027	0.231∗∗∗	−0.164∗∗	0.291∗∗∗	—

1. Anxiety; 2. Psychological openness; 3. Help-seeking propensity; 4. Indifference to stigma; 5. Stigma to others; 6. Social distance; 7. Anticipated stigma; 8. Self-stigma; 9. Knowledge of mental health problems; 10. Stereotypes; 11. Help-seeking and first aid skills; 12. Self-help strategies. *p < 0.05; **p < 0.01; ***p < 0.001.

A greater willingness to seek help was associated with enhanced mental health knowledge [*r*(349) = 0.309, *p* < 0.001], increased social distance [*r*(349) = 0.358, *p* < 0.001], stronger help-related skills [*r*(349) = 0.619, *p* < 0.001], and more frequent use of self-help methods [*r*(349) = 0.277, *p* < 0.001]. Conversely, it was negatively related to different forms of stigma such as stigma to others [*r*(349) = −0.226, *p* < 0.001] and stereotypical beliefs [*r*(349) = −0.222, *p* < 0.001].

Social distance was positively connected with mental health knowledge [*r*(349) = 0.332, *p* < 0.001] and help-seeking skills [*r*(349) = 0.213, *p* < 0.001], but negatively with self-stigma [*r*(349) = −0.331, *p* < 0.001] and stereotypical views [*r*(349) = −0.171, *p* = 0.001]. Notably, anticipated stigma and self-stigma demonstrated a strong mutual relationship [*r*(349) = 0.824, *p* < 0.001]. Stereotypical attitudes were inversely related to help-seeking skills [*r*(349) = −0.138, *p* = 0.009] and self-help engagement [*r*(349) = −0.164, *p* = 0.002], which were themselves positively interconnected [*r*(349) = 0.291, *p* < 0.001].

### Mediation analyses

It is important to note that due to the cross-sectional design of this study, the temporal direction between anxiety, mental health literacy, stigma, and attitudes toward seeking care cannot be definitively determined. The following mediation models reflect indirect relationships consistent with our theoretical framework rather than causal pathways.

#### Psychological openness

**Figure 2:**
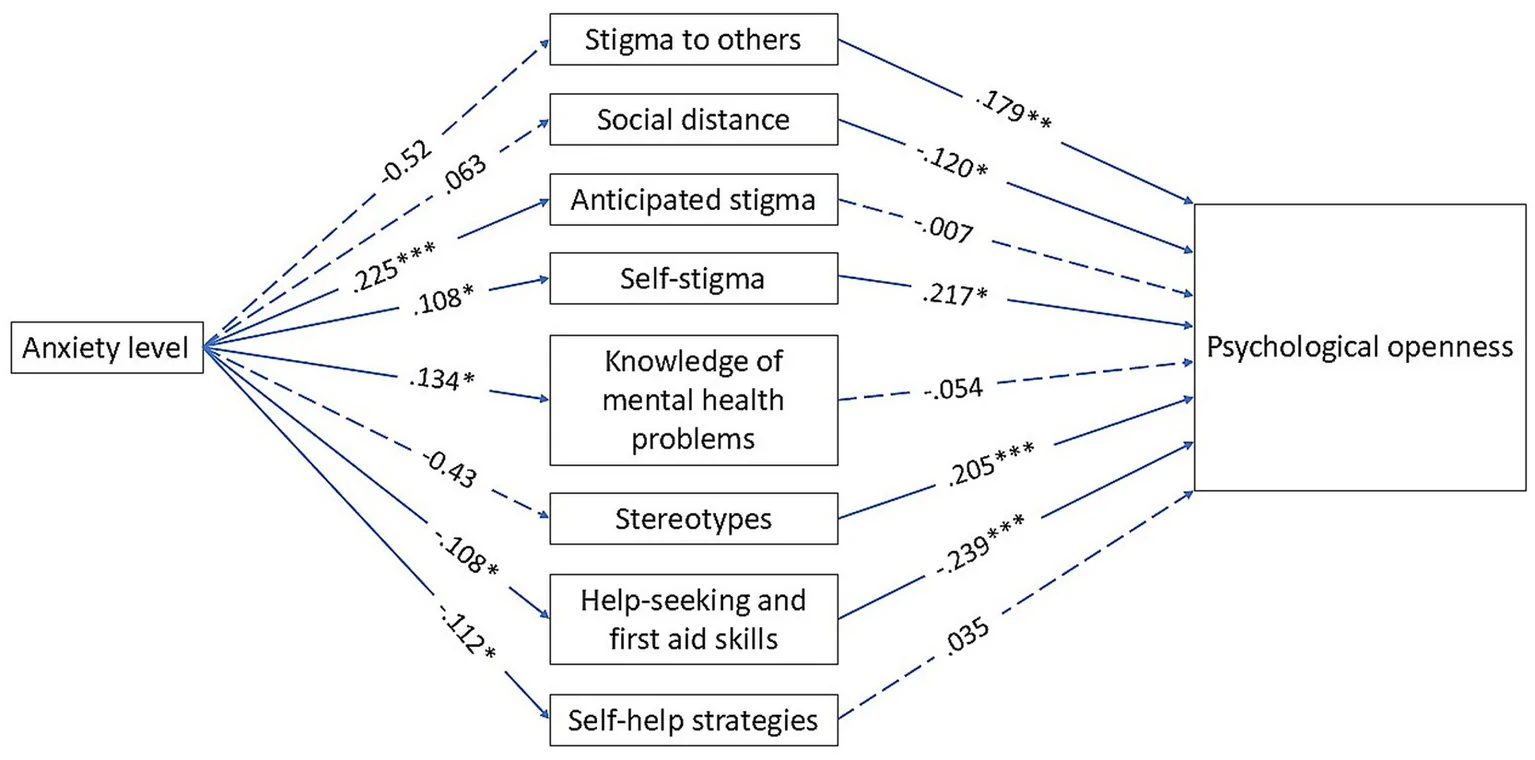
Empirical path diagram for the parallel mediation model predicting psychological openness. Path coefficients represent standardized estimates (β). Solid arrows indicate significant paths (*p<0.05, **p<0.01, *p*<0.001). Dashed arrows indicate non-significant paths.

The model explained 38.0% of the variance in psychological openness (*R^2^* = 0.380). Anxiety level demonstrated a significant positive association with psychological openness (*β* = 0.096, *p* = 0.036), alongside stigma toward others (*β* = 0.179, *p* = 0.001), self-stigma (*β* = 0.216, *p* = 0.010), and stereotypes (*β* = 0.205, *p* < 0.001). In contrast, social distance (*β* = −0.120, *p* = 0.019) and help-seeking and first aid skills (*β* = −0.239, *p* < 0.001) showed significant negative associations. None of the examined indirect pathways reached statistical significance, indicating that the mediators included in the model did not account for the relationship between anxiety and psychological openness. Consistent with the bivariate correlation (*r* = 0.107, *p* = 0.044), both the direct path (*β* = 0.096, *p* = 0.036) and the total association (*β* = 0.107, *p* = 0.044) of anxiety on psychological openness were statistically significant. These findings suggest that anxiety is directly associated with greater psychological openness, while the selected mediators do not contribute meaningfully to explaining this relationship. The detailed results of the mediation analysis are presented in Table 4. The complete empirical path diagram for this parallel mediation model, illustrating all standardized path coefficients and their statistical significance, is presented in Figure 2.

**Table 4:** Indirect and total effects on psychological openness.

Type	Effect	Estimate	SE	95% CI [LL, UL]	*β*	*p*
Indirect	ANX ⇒ SO ⇒ PO	−0.015	0.016	[−0.046, 0.016]	−0.009	0.349
	ANX ⇒ SD ⇒ PO	−0.012	0.012	[−0.036, 0.012]	−0.008	0.291
	ANX ⇒ AS ⇒ PO	−0.002	0.029	[−0.059, 0.055]	−0.002	0.931
	ANX ⇒ SS ⇒ PO	0.038	0.024	[−0.009, 0.085]	0.023	0.110
	ANX ⇒ KMHP ⇒ PO	−0.012	0.012	[−0.036, 0.012]	−0.007	0.317
	ANX ⇒ ST ⇒ PO	−0.014	0.018	[−0.049, 0.021]	−0.009	0.433
	ANX ⇒ HSFAS ⇒ PO	0.041	0.022	[−0.002, 0.084]	0.026	0.059
	ANX ⇒ SHS ⇒ PO	−0.006	0.009	[−0.024, 0.012]	−0.004	0.475
Direct	ANX ⇒ PO	0.154	0.073	[0.011, 0.297]	0.096	0.036
Total	ANX ⇒ PO	0.172	0.085	[0.005, 0.339]	0.107	0.044
Component	ANX ⇒ SO	−0.057	0.058	[−0.171, 0.057]	−0.052	0.329
	SO ⇒ PO	0.266	0.081	[0.107, 0.425]	0.179	0.001
	ANX ⇒ SD	0.061	0.051	[−0.039, 0.161]	0.063	0.238
	SD ⇒ PO	−0.200	0.085	[−0.367, −0.033]	−0.120	0.019
	ANX ⇒ AS	0.299	0.069	[0.164, 0.434]	0.225	<0.001
	AS ⇒ PO	−0.008	0.096	[−0.196, 0.180]	−0.007	0.931
	ANX ⇒ SS	0.140	0.069	[0.005, 0.275]	0.108	0.042
	SS ⇒ PO	0.269	0.104	[0.065, 0.473]	0.217	0.010
	ANX ⇒ KMHP	0.120	0.048	[0.026, 0.214]	0.134	0.012
	KMHP ⇒ PO	−0.097	0.089	[−0.271, 0.077]	−0.054	0.275
	ANX ⇒ ST	−0.025	0.032	[−0.088, 0.038]	−0.043	0.425
	ST ⇒ PO	0.554	0.132	[0.295, 0.813]	0.205	<0.001
	ANX ⇒ HSFAS	−0.058	0.028	[−0.113, −0.003]	−0.108	0.042
	HSFAS ⇒ PO	−0.718	0.138	[−0.988, −0.448]	−0.239	<0.001
	ANX ⇒ SHS	−0.067	0.031	[−0.128, −0.006]	−0.112	0.034
	SHS ⇒ PO	0.096	0.127	[−0.153, 0.345]	0.035	0.448

Confidence intervals computed with method: Standard (Delta method). LL, Lower Limit; UL, Upper Limit. Betas are completely standardized effect sizes. ANX, Anxiety; SO, Stigma to Others; PO, Psychological openness; SD, Social Distance; AS, Anticipated Stigma; SS, Self-Stigma; KMHP, Knowledge of mental health problems; ST, Stereotypes; HSFAS, Help-seeking and first aid skills; SHS, Self-Help Strategies.

#### Help-seeking propensity

**Figure 3:**
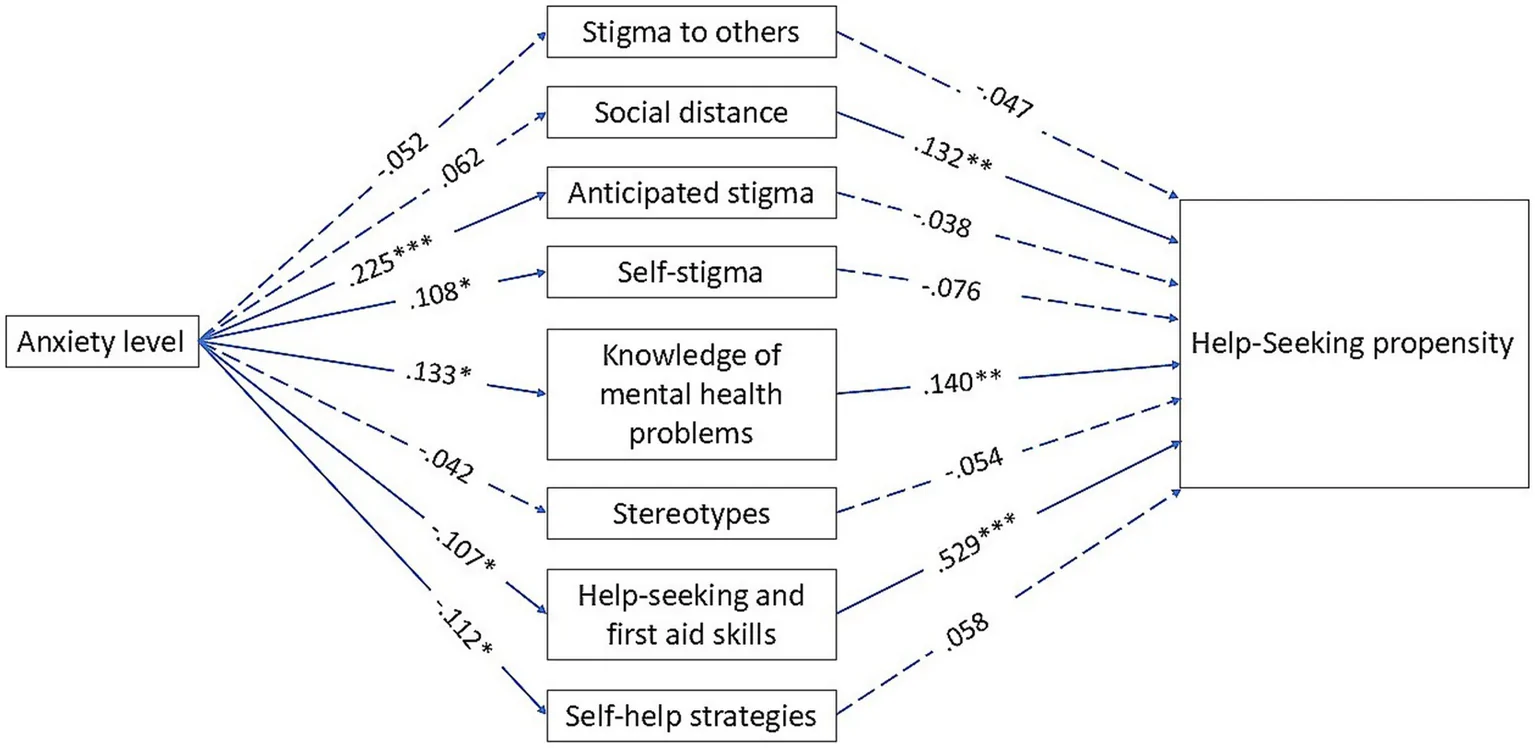
Empirical path diagram for the parallel mediation model predicting help-seeking propensity. Path coefficients represent standardized estimates (β). Solid arrows indicate significant paths (*p<0.05, **p<0.01, *p*<0.001). Dashed arrows indicate non-significant paths.

The model explained 51.3% of the variance in help-seeking propensity (*R^2^* = 0.513). The significant variables associated with the help-seeking propensity criterion are: social distance (*β* = 0.132, *p* = 0.004), knowledge of mental health problems (*β* = 0.140, *p* = 0.002), and help-seeking and first aid skills (*β* = 0.529, *p* < 0.001). Among the indirect relationships, the path from anxiety levels to help-seeking propensity through knowledge of mental health problems is significant and positive (*β* = 0.018, *p* = 0.048) and significant and negative through help-seeking and first aid skills (*β* = −0.057, *p* = 0.045). The total association is negative and significant (*β* = −0.120, *p* = 0.024). The results can be seen in Table 5. The complete empirical path diagram for the parallel mediation model predicting Help-Seeking Propensity is visually summarized in Figure 3.

**Table 5:** Indirect and total effects on help-seeking propensity.

Type	Effect	Estimate	SE	95% CI [LL, UL]	*β*	*p*
Indirect	ANX ⇒ SO ⇒ HSP	0.003	0.005	[−0.007, 0.013]	0.002	0.489
	ANX ⇒ SD ⇒ HSP	0.011	0.010	[−0.009, 0.031]	0.008	0.274
	ANX ⇒ AS ⇒ HSP	−0.012	0.022	[−0.055, 0.031]	−0.008	0.585
	ANX ⇒ SS ⇒ HSP	−0.011	0.012	[−0.035, 0.013]	−0.008	0.359
	ANX ⇒ KMHP ⇒ HSP	0.026	0.013	[0.001, 0.051]	0.018	0.048
	ANX ⇒ ST ⇒ HSP	0.003	0.004	[−0.005, 0.011]	0.002	0.501
	ANX ⇒ HSFAS ⇒ HSP	−0.080	0.040	[−0.158, −0.002]	−0.057	0.045
	ANX ⇒ SHS ⇒ HSP	−0.009	0.007	[−0.023, 0.005]	−0.006	0.240
Direct	ANX ⇒ HSP	−0.100	0.057	[−0.212, 0.012]	−0.071	0.080
Total	ANX ⇒ HSP	−0.169	0.074	[−0.314, −0.024]	−0.120	0.024
Component	ANX ⇒ SO	−0.056	0.057	[−0.168, 0.056]	−0.052	0.329
	SO ⇒ HSP	−0.062	0.063	[−0.185, 0.061]	−0.047	0.327
	ANX ⇒ SD	0.060	0.051	[−0.040, 0.160]	0.062	0.238
	SD ⇒ HSP	0.192	0.066	[0.063, 0.321]	0.132	0.004
	ANX ⇒ AS	0.299	0.069	[0.164, 0.434]	0.225	<0.001
	AS ⇒ HSP	−0.041	0.074	[−0.186, 0.104]	−0.038	0.582
	ANX ⇒ SS	0.140	0.068	[0.007, 0.273]	0.108	0.042
	SS ⇒ HSP	−0.083	0.081	[−0.242, 0.076]	−0.076	0.304
	ANX ⇒ KMHP	0.120	0.047	[0.028, 0.212]	0.133	0.012
	KMHP ⇒ HSP	0.220	0.069	[0.085, 0.355]	0.140	0.002
	ANX ⇒ ST	−0.025	0.031	[−0.086, 0.036]	−0.042	0.425
	ST ⇒ HSP	−0.129	0.103	[−0.331, 0.073]	−0.054	0.210
	ANX ⇒ HSFAS	−0.057	0.028	[−0.112, −0.002]	−0.107	0.042
	HSFAS ⇒ HSP	1.395	0.108	[1.183, 1.607]	0.529	<0.001
	ANX ⇒ SHS	−0.066	0.031	[−0.127, −0.005]	−0.112	0.034
	SHS ⇒ HSP	0.140	0.099	[−0.054, 0.334]	0.058	0.158

Confidence intervals computed with method: Standard (Delta method). LL, Lower Limit; UL, Upper Limit. Betas are completely standardized effect sizes. ANX, Anxiety; SO, Stigma to Others; HSP, Help-Seeking Propensity; SD, Social Distance; AS, Anticipated Stigma; SS, Self-Stigma; KMHP, Knowledge of mental health problems; ST, Stereotypes; HSFAS, Help-seeking and first aid skills; SHS, Self-Help Strategies.

Regarding the Indifference to Stigma dimension, no mediation analysis was performed because the preliminary requirements for such a model were not met. Specifically, bivariate correlation analysis showed that anxiety was not significantly associated with indifference to stigma (*r* = 0.047, *p* = 0.376). Given the absence of a direct relationship between transient anxiety and the outcome variable, this dimension was excluded from the mediation testing to maintain the statistical integrity and parsimony of the final model.

## Discussion

The present study examined the relationships between transient anxiety levels and attitudes toward professional psychological help-seeking, as well as the mediating roles of mental health literacy and stigma-related processes. Overall, the findings provided partial support for the proposed hypotheses and indicated that transient anxiety was associated with help-seeking attitudes through multiple, and at times opposing, cognitive and affective pathways. In particular, experiencing transient anxiety symptoms appeared to relate to the recognition of psychological difficulties while simultaneously activating processes that may hinder the translation of awareness into actual help-seeking behavior.

The first hypothesis, which proposed a strong positive association between anxiety and attitudes toward help-seeking, was only partially supported. Transient anxiety levels were positively associated with psychological openness, suggesting that individuals experiencing acute psychological distress were more likely to acknowledge psychological difficulties. However, anxiety demonstrated a statistically negative association with help-seeking propensity, and no association was observed with indifference to stigma. Rather than a mere negative trend, this significant inverse relationship indicates that higher levels of transient anxiety are actually associated to a reduced readiness to actively seek professional help.

From a theoretical perspective, these findings are consistent with models of help-seeking that distinguished between problem recognition and action initiation (Rickwood et al., 2007). Transient anxiety appears to enhance awareness and acknowledgment of distress, yet this heightened awareness does not necessarily translate into behavioral intention. This pattern is in line with the Theory of Planned Behavior, which posits that attitudes alone are insufficient to account for behavior in the absence of perceived control and supportive social norms (Ajzen, 1985). Importantly, rather than directly reflecting the entrenched avoidance mechanisms that characterize clinical anxiety disorders and drive the documented six-year treatment delay (Clement et al., 2015; Johnson and Coles, 2013), our findings capture a distinct, pre-clinical phenomenon within the general population. Here, the acute discomfort of transient anxiety operates within an early window of opportunity: it successfully increases psychological openness, yet this emotional catalyst fails to translate into action because it simultaneously exposes a deficit in practical help-seeking skills. Therefore, our results suggest that the bottleneck in the help-seeking pipeline for non-clinical individuals is not yet clinical avoidance, but a structural lack of perceived capacity to navigate the system, highlighting a critical point for early preventive intervention before symptoms chronicize. Anxiety-related avoidance, fear of negative evaluation, and uncertainty regarding treatment outcomes may therefore inhibit action even when psychological openness is increased (Clement et al., 2015; Johnson and Coles, 2013).

From a practical standpoint, these results suggested that interventions focusing exclusively on symptom awareness may be insufficient. While individuals experiencing transient anxiety may recognize their need for help, additional emotional, cognitive, and social barriers must be addressed to facilitate engagement with professional mental health services (Gulliver et al., 2010).

The second hypothesis proposed a positive relationship between anxiety levels and mental health literacy. This hypothesis was partially supported. Transient anxiety was positively associated with knowledge of mental health problems but negatively associated with help-seeking and first aid skills, as well as with self-help strategies.

These findings suggested that general psychological distress is selectively related to different components of mental health literacy. Heightened transient anxiety may increase vigilance and information-seeking tendencies, which could explain its association with greater recognition and knowledge of mental health problems (Hartley and Phelps, 2012). At the same time, acute emotional distress may undermine confidence in one’s ability to manage distress or navigate help-seeking options, resulting in lower perceived competence and reduced engagement in self-help strategies (Gulliver et al., 2010). This differentiated pattern supports the view that mental health literacy is a multidimensional construct whose components are differentially related to emotional states (Jorm, 2000; Calear et al., 2021).

From a practical perspective, these results highlight the limitations of purely informational psychoeducational approaches. Increasing knowledge alone may not be sufficient to promote adaptive help-seeking if individuals do not feel capable of acting on that knowledge. Effective interventions should therefore integrate informational content with structured opportunities for skill development.

The third hypothesis, which proposed that mental health literacy would mediate the relationship between anxiety and help-seeking attitudes, was supported through two opposing indirect pathways. Transient anxiety demonstrated a positive indirect association with help-seeking propensity through increased knowledge of mental health problems, while simultaneously demonstrating a negative indirect association through reduced help-seeking and first aid skills.

These contrasting pathways underscore the complex and multidimensional role of mental health literacy. While increased knowledge may facilitate recognition of the need for professional help and may enhance motivation to seek support (Coles and Coleman, 2010; Yang et al., 2023), reduced perceived competence may counteract this association by limiting individuals’ confidence in their ability to engage with mental health services. However, it is crucial to interpret these findings within the context of partial mediation. Given that our mediation models accounted for 51.3% of the variance in help-seeking propensity and 38% in psychological openness, mental health literacy and practical help-seeking skills represent just one set of pathways among several. Other unmeasured factors such as structural healthcare barriers, financial constraints, or additional psychological mechanisms likely play equally important roles in explaining the broader treatment gap. This dual mechanism helps explain a significant proportion of why transient anxiety does not consistently relate to help-seeking behavior despite increasing awareness of psychological difficulties, while acknowledging that it is not the sole explanation.

From a theoretical standpoint, these findings significantly enrich the Theory of Planned Behavior (TPB) and the ABC model of attitudes by moving beyond a simple categorization of variables to reveal a profound structural conflict within the help-seeking process. When mapped onto the ABC model, mental health knowledge represents the cognitive component, stigma acts as the affective component, and help-seeking skills constitute the behavioral component. Within the TPB framework, these elements find a natural alignment: public and anticipated stigma represent the perceived social pressures of subjective norms, while help-seeking skills directly underpin perceived behavioral control (PBC). By conceptualizing our mediation findings through this integrated lens, it becomes evident that transient anxiety functions as a fragmented psychological force. It acts as an affective and cognitive catalyst that successfully fosters a positive attitude (manifested as psychological openness), but simultaneously undermines the behavioral dimension by eroding confidence in help-seeking skills (PBC). Framing these findings as a structural conflict between a favorable attitudinal disposition and a deficient sense of behavioral control provides a sophisticated theoretical explanation for why emotional distress so frequently fails to generate actual behavioral intention (help-seeking propensity) in non-clinical populations.

From a practical perspective, these results reinforce the necessity of moving beyond traditional, information-only psychoeducation, but they must not be misinterpreted as an invitation to neglect core attitudinal interventions. Grounded in the Mental Health First Aid (MHFA) framework, mental health knowledge, stigma reduction, and practical navigation skills must be treated as inseparable, synergistic pillars where one cannot effectively function without the others. While interventions must actively integrate structured skill-building components to ensure that psychological openness successfully translates into intention, this approach should complement, not replace, literacy and anti-stigma efforts. For instance, programs modeled after MHFA should teach individuals the “active navigation” of the healthcare system—such as how to initiate professional contact—while systematically reducing the surrounding stigma.

Furthermore, our findings regarding the negative link between anxiety and skills warrant a granular interpretation: the observed erosion of skills under distress likely reflects a drop in the perceived efficiency (self-efficacy) of those skills when experiencing acute anxiety, rather than a decline in the structural willingness to use them. Highly anxious individuals do not necessarily reject the utility of help-seeking pathways; rather, their immediate psychological distress temporarily compromises their confidence to effectively execute these tasks. Consequently, because anxiety is also associated with higher anticipated stigma, interventions should incorporate “social contact” strategies, such as lived-experience narratives from peers. This holistic approach normalizes the process, reinforces self-efficacy under stress, and counteracts the cognitive disengagement often observed in highly anxious individuals, offering culturally relevant strategies essential for the Romanian context to bridge the gap between acknowledging distress and initiating specialized care.

The fourth hypothesis, which anticipated a positive association between anxiety and stigma, was supported for anticipated stigma and self-stigma. Individuals with higher transient anxiety levels reported stronger expectations of negative social judgment and higher levels of internalized stigma.

These findings are consistent with previous research indicating that general emotional distress is associated with heightened sensitivity to social evaluation and negative self-appraisals (Ociskova et al., 2015; Fox et al., 2018). Transient anxiety may amplify concerns about being judged by others and increase the likelihood of internalizing stigmatizing beliefs, thereby creating additional psychological barriers to help-seeking (Corrigan and Watson, 2002; Clement et al., 2015).

From a clinical perspective, these results suggest that interventions targeting individuals experiencing general psychological distress individuals should explicitly address anticipated and self-stigma, as these processes may exacerbate avoidance tendencies and reduce willingness to engage with professional mental health services.

Regarding the indifference to stigma dimension, the data did not support the implementation of a mediation model. The absence of a significant bivariate correlation between anxiety levels and indifference to stigma suggests that the degree to which individuals disregard social judgment is a relatively stable construct, functioning independently of their current emotional distress. Theoretically, this lack of association implies that indifference to stigma may act more as a baseline personality trait or a deeply internalized cultural attitude rather than a reactive psychological state related to anxiety symptoms (Vogel et al., 2007; Mackenzie et al., 2004). In the Romanian context, where social evaluation remains a documented barrier to care, these findings indicate that an escalation in transient anxiety may heighten the awareness of distress without necessarily buffering the individual against the perceived weight of social judgment.

Furthermore, because indifference to stigma captures a cognitive detachment from public perceptions, its independence from anxiety levels suggests that interventions targeting symptom reduction alone will likely not shift this specific attitudinal component (Eisenberg et al., 2009). The lack of statistical grounds for a robust mediation led to the conclusion that, within this sample, indifference to stigma functions as an autonomous factor in the help-seeking process rather than a mechanism associated with anxiety-related distress. Consequently, this dimension was excluded from the final mediation testing to preserve the statistical integrity of the analysis, highlighting that efforts to foster resilience toward stigma must be addressed through specialized attitudinal interventions rather than symptom-focused psychoeducation (Corrigan, 2016).

### Practical implications

Beyond theoretical insights, these results suggest a potential direction for enhancing and necessitate a shift in how psychoeducational interventions are structured within the Romanian mental health landscape. Since psychological openness alone does not appear to guarantee a transition to specialized care, programs might benefit from integrating concrete skill-building modules designed to foster help-seeking self-efficacy. A robust framework for this can be inspired by Mental Health First Aid (MHFA), which moves beyond symptom recognition to equip individuals with “active navigation” competencies, such as the ability to initiate professional contact and manage the logistical nuances of clinical intake (Kitchener and Jorm, 2008; Bond et al., 2015). However, given the cross-sectional nature of these data, it is important to emphasize that improving these practical skills alone is unlikely to single-handedly resolve broader treatment delays.

Furthermore, addressing the anticipated stigma identified in this sample may require more than purely informational strategies. Evidence suggests that “social contact” interventions, which utilize first-person narratives of treatment navigation, are significantly more effective at dismantling behavioral avoidance than traditional educational materials (Corrigan et al., 2012; Wood and Wahl, 2006). In practice, exploring approaches like peer-led digital storytelling can provide a tangible “roadmap” for the therapeutic process, potentially helping to demystify the clinical encounter and mitigate the cognitive disengagement often triggered by high levels of anxiety. By pivoting from diagnostic literacy to functional navigation, these strategies target key psychological barriers, though further longitudinal research is necessary to confirm their independent effectiveness in translating psychological distress into actual behavioral action.

### Limitations

Several methodological constraints must be acknowledged regarding the present research. Primarily, the cross-sectional nature of the study design represents a significant limitation, as it precludes the establishment of causal trajectories between the variables. Because data were captured at a single point in time, the observed relationships between anxiety, mental health literacy, and stigma-related attitudes remain non-causal. Consequently, it cannot be definitively determined whether fluctuations in anxiety levels precede changes in help-seeking propensity or if these associations are influenced by unmeasured confounding factors.

Furthermore, the generalizability of these findings is constrained by the use of convenience sampling within a specific Romanian context. Because we utilized a convenience sample from the general population and measured anxiety using the DASS-21—a screening tool rather than a clinical diagnostic instrument—the present study does not investigate clinical disorders. Therefore, our conclusions reflect mechanisms associated with general psychological distress and transient anxiety symptoms, rather than specific clinical anxiety disorders. The sample consisted predominantly of residents from urban environments and was notably skewed toward female participants. This demographic distribution may reflect specific socio-cultural attitudes that are not necessarily representative of the broader Romanian population or more diverse social environments. Additionally, the reliance on self-report instruments introduces the potential for social desirability bias, as participants may have altered their responses regarding stigma or psychological openness to align with perceived social norms rather than disclosing their genuine perspectives.

### Further directions

To address the constraints of the current research, future investigations should prioritize longitudinal designs capable of tracking individual psychological trajectories over extended periods. Such studies are necessary to clarify the temporal precedence between anxiety symptoms and attitudinal shifts, specifically testing whether changes in distress levels directly cause fluctuations in help-seeking propensity. Furthermore, exploring the role of specific anxiety subtypes could provide a more granular understanding of how different manifestations of distress interact with mental health literacy components.

Additionally, future studies should include clinical samples to investigate whether the multidimensional pathways identified in this study operate similarly among individuals with formally diagnosed anxiety disorders. Also future research should expand the demographic scope by employing stratified sampling techniques to include more diverse populations, such as individuals from rural areas or older age groups, ensuring a broader representation of the Romanian socio-cultural landscape. Finally, integrating qualitative or mixed-methods approaches would be beneficial for capturing the subjective, lived experiences of those navigating the mental health system. This would allow for the identification of nuanced barriers and facilitators that quantitative scales might overlook, providing a more comprehensive framework for developing culturally tailored interventions.

## Conclusion

In this research, we examined how levels of anxiety related to individuals’ willingness to seek professional psychological support, using a sample of Romanianconvenience sample. Our approach focused on understanding the indirect roles played by mental health stigma and various aspects of mental health literacy. These aspects included the recognition of psychological symptoms, awareness of risk factors and causes, understanding of self-help and professional treatment options, attitudes toward seeking help, and the ability to access mental health information.

These findings point to the need for mental health programs that do more than raise awareness. While anxiety is associated with enhanced psychological openness, it demonstrates a statistically significant negative association with help-seeking propensity and relates to stigma-related attitudes through both cognitive and emotional mediators. Efforts must also build self-efficacy by teaching practical skills to navigate the help-seeking process. Interventions should also target internalized forms of stigma, which appear to play a central role in shaping both attitudes and behaviors toward help-seeking. Promoting accurate understanding of mental illness is important, but it must be paired with opportunities to build confidence and reduce distancing.

In conclusion, anxiety is associated with how people perceive, act, and emotionally respond to mental health problems not in a uniform way, but through distinct and sometimes conflicting routes. Recognizing these pathways is essential for designing interventions that are both psychologically sensitive and behaviorally effective.

## Data Availability

The raw data supporting the conclusions of this article will be made available by the authors, without undue reservation.

## References

[ref1] Ajzen I. (1985). “From intentions to Actions: A Theory of Planned Behavior,” in Action Control. SSSP Springer Series in Social Psychology, eds. Kuhl J. Beckmann J. (Berlin, Heidelberg: Springer).

[ref2] Ajzen I. (2020). The theory of planned behavior: frequently asked questions. Hum. Behav. Emerg. Technol. 2, 314–324. doi: 10.1002/hbe2.195

[ref3] Ajzen I. Fishbein M. (2000). Attitudes and the attitude-behavior relation: reasoned and automatic processes. Eur. Rev. Soc. Psychol. 11, 1–33. doi: 10.1080/14792779943000116

[ref4] Al-Shannaq Y. Aldalaykeh M. (2023). Suicide literacy, suicide stigma, and psychological help seeking attitudes among Arab youth. Curr. Psychol. 42, 6532–6544. doi: 10.1007/s12144-021-02007-9, 34177209 PMC8214717

[ref5] Andrews G. Henderson S. (2000). Unmet need in Psychiatry: Problems, Resources, Responses. Cambridge: Cambridge University Press.

[ref6] Aromaa E. Tolvanen A. Tuulari J. Wahlbeck K. (2011). Personal stigma and use of mental health services among people with depression in a general population in Finland. BMC Psychiatry 11:52. doi: 10.1186/1471-244X-11-52, 21453504 PMC3079625

[ref7] Baldwin D. S. Anderson I. M. Nutt D. J. Allgulander C. Bandelow B. den Boer J. A. . (2014). Evidence-based pharmacological treatment of anxiety disorders, post-traumatic stress disorder and obsessive-compulsive disorder: a revision of the 2005 guidelines from the British Association for Psychopharmacology. J. Psychopharmacol. (Oxford, England) 28, 403–439. doi: 10.1177/0269881114525674, 24713617

[ref8] Bond K. S. Jorm A. F. Kitchener B. A. Reavley N. J. (2015). Mental health first aid training for Australian medical and nursing students: an evaluation study. BMC Psychol. 3:11. doi: 10.1186/s40359-015-0069-0, 25914827 PMC4399395

[ref9] Boyd J. E. Adler E. P. Otilingam P. G. Peters T. (2014). Internalized stigma of mental illness (ISMI) scale: a multinational review. Compr. Psychiatry 55, 221–231. doi: 10.1016/j.comppsych.2013.06.005, 24060237

[ref10] Breckler S. J. (1984). Empirical validation of affect, behavior, and cognition as distinct components of attitude. J. Pers. Soc. Psychol. 47, 1191–1205. doi: 10.1037//0022-3514.47.6.1191, 6527214

[ref11] Calear A. L. Batterham P. J. Torok M. McCallum S. (2021). Help-seeking attitudes and intentions for generalised anxiety disorder in adolescents: the role of anxiety literacy and stigma. Eur. Child Adolesc. Psychiatry 30, 243–251. doi: 10.1007/s00787-020-01512-9, 32180026

[ref12] Campos L. Dias P. Costa M. Rabin L. Miles R. Lestari S. . (2022). Mental health literacy questionnaire-short version for adults (MHLq-SVa): validation study in China, India, Indonesia, Portugal, Thailand, and the United States. BMC Psychiatry 22:713. doi: 10.1186/s12888-022-04308-0, 36384505 PMC9668212

[ref13] Carpenter J. K. Andrews L. A. Witcraft S. M. Powers M. B. Smits J. A. J. Hofmann S. G. (2018). Cognitive behavioral therapy for anxiety and related disorders: a meta-analysis of randomized placebo-controlled trials. Depress. Anxiety 35, 502–514. doi: 10.1002/da.22728, 29451967 PMC5992015

[ref14] Chen J. (2018). Some people may need it, but not me, not now: seeking professional help for mental health problems in urban China. Transcult. Psychiatry 55, 754–774. doi: 10.1177/1363461518792741 30113276

[ref15] Clement S. Schauman O. Graham T. Maggioni F. Evans-Lacko S. Bezborodovs N. . (2015). What is the impact of mental health-related stigma on help-seeking? A systematic review of quantitative and qualitative studies. Psychol. Med. 45, 11–27. doi: 10.1017/S0033291714000129, 24569086

[ref16] Coles M. E. Coleman S. L. (2010). Barriers to treatment seeking for anxiety disorders: initial data on the role of mental health literacy. Depress. Anxiety 27, 63–71. doi: 10.1002/da.20620, 19960488

[ref9001] Corrigan P. (2004). How stigma interferes with mental health care. American Psychologist, 59, 614–625. doi: 10.1037/0003-066X.59.7.614 15491256

[ref17] Corrigan P. W. (2016). The Stigma of Disease and Disability. Washington, DC: American Psychological Association.

[ref18] Corrigan P. W. Morris S. B. Michaels P. J. Rafacz J. D. Rüsch N. (2012). Challenging the public stigma of mental illness: a meta-analysis of outcome studies. Psychiatr. Serv. 63, 963–973. doi: 10.1176/appi.ps.201100529, 23032675

[ref19] Corrigan P. Thompson V. Lambert D. Sangster Y. Noel J. G. Campbell J. (2003). Perceptions of discrimination among persons with serious mental illness. Psychiatr. Serv. (Wash. D.C.) 54, 1105–1110. doi: 10.1176/appi.ps.54.8.1105 12883137

[ref9002] Corrigan P. W. Shapiro J. R. (2010). Measuring the impact of programs that challenge the public stigma of mental illness. Clinical Psychology Review, 30, 907–922. doi: 10.1016/j.cpr.2010.06.004 20674114 PMC2952670

[ref20] Corrigan P. W. Watson A. C. (2002). The paradox of self-stigma and mental illness. Clin. Psychol. Sci. Pract. 9, 35–53. doi: 10.1093/clipsy.9.1.35

[ref21] Curcio C. Corboy D. (2020). Stigma and anxiety disorders: a systematic review. Stigma Health 5, 125–137. doi: 10.1037/sah0000183

[ref22] Docksey A. E. Gray N. S. Davies H. B. Simkiss N. Snowden R. J. (2022). The stigma and self-stigma scales for attitudes to mental health problems: psychometric properties and its relationship to mental health problems and absenteeism. Health Psychol. Res. 10:35630. doi: 10.52965/001c.35630, 35928586 PMC9346955

[ref23] Eisenberg D. Downs M. F. Golberstein E. Zivin K. (2009). Stigma and help seeking for mental health among college students. Med. Care Res. Rev. 66, 522–541. doi: 10.1177/1077558709335173, 19454625

[ref24] Fischer E. H. Turner J. I. (1970). Orientations to seeking professional help: development and research utility of an attitude scale. J. Consult. Clin. Psychol. 35, 79–90. doi: 10.1037/h0029636, 5487612

[ref25] Fox A. B. Smith B. N. Vogt D. (2018). How and when does mental illness stigma impact treatment seeking? Longitudinal examination of relationships between anticipated and internalized stigma, symptom severity, and mental health service use. Psychiatry Res. 268, 15–20. doi: 10.1016/j.psychres.2018.06.036, 29986172

[ref26] GBD 2019 Diseases and Injuries Collaborators (2020). Global burden of 369 diseases and injuries in 204 countries and territories, 1990-2019: a systematic analysis for the global burden of disease study 2019. Lancet 396, 1204–1222. doi: 10.1016/S0140-6736(20)30925-9, 33069326 PMC7567026

[ref27] Gulliver A. Griffiths K. M. Christensen H. (2010). Perceived barriers and facilitators to mental health help-seeking in young people: a systematic review. BMC Psychiatry 10:113. doi: 10.1186/1471-244X-10-113, 21192795 PMC3022639

[ref28] Han M. Cha R. Lee H. A. Lee S. E. (2017). Mental-illness stigma among Korean immigrants: role of culture and destigmatization strategies. Asian Am. J. Psychol. 8, 134–141. doi: 10.1037/aap0000074

[ref29] Hartley C. A. Phelps E. A. (2012). Anxiety and decision-making. Biol. Psychiatry 72, 113–118. doi: 10.1016/j.biopsych.2011.12.027, 22325982 PMC3864559

[ref30] Heinig I. Wittchen H. U. Knappe S. (2021). Help-seeking behavior and treatment barriers in anxiety disorders: results from a representative German community survey. Community Ment. Health J. 57, 1505–1517. doi: 10.1007/s10597-020-00767-5, 33471256 PMC8531057

[ref31] Hohn A. A. Maricutoiu L. (2024). Help seeking behaviours in anxiety disorders: a systematic scoping review. J. Evid. Based Psychother. 24, 63–80. doi: 10.24193/jebp.2024.1.4

[ref32] Hu L.-T. Bentler P. M. (1999). Cutoff criteria for fit indexes in covariance structure analysis: conventional criteria versus new alternatives. Struct. Equ. Model. 6, 1–55. doi: 10.1080/10705519909540118, 37339054

[ref33] Johnson E. M. Coles M. E. (2013). Failure and delay in treatment-seeking across anxiety disorders. Community Ment. Health J. 49, 668–674. doi: 10.1007/s10597-012-9543-9, 23054147

[ref34] Jorm A. F. (2000). Mental health literacy: public knowledge and beliefs about mental disorders. Br. J. Psychiatry 177, 396–401. doi: 10.1192/bjp.177.5.396, 11059991

[ref35] Jorm A. F. Korten A. E. Jacomb P. A. Christensen H. Rodgers B. Pollitt P. (1997). "mental health literacy": a survey of the public's ability to recognise mental disorders and their beliefs about the effectiveness of treatment. Med. J. Aust. 166, 182–186. doi: 10.5694/j.1326-5377.1997.tb140071.x, 9066546

[ref36] Jung H. Von Sternberg K. Davis K. (2017). The impact of mental health literacy, stigma, and social support on attitudes toward mental health help-seeking. Int. J. Ment. Health Promot. 19, 252–267. doi: 10.1080/14623730.2017.1345687

[ref37] Kaiser F. G. Wilson M. (2019). The Campbell paradigm as a behavior-predictive reinterpretation of the classical tripartite model of attitudes. Eur. Psychol. 24, 359–374. doi: 10.1027/1016-9040/a000364, 32116425 PMC7039345

[ref38] Keum B. T. Hill C. E. Kivlighan D. M. Lu Y. (2018). Group- and individual-level self-stigma reductions in promoting psychological help-seeking attitudes among college students in helping skills courses. J. Couns. Psychol. 65, 661–668. doi: 10.1037/cou0000283, 30035590

[ref39] Kitchener B. A. Jorm A. F. (2008). Mental health first aid: an international programme for early intervention. Early Interv. Psychiatry 2, 55–61. doi: 10.1111/j.1751-7893.2007.00056.x, 21352133

[ref40] Link B. G. Phelan J. C. (2001). Conceptualizing stigma. Annu. Rev. Sociol. 27, 363–385. doi: 10.1146/annurev.soc.27.1.363

[ref41] Lovibond P. F. Lovibond S. H. (1995). The structure of negative emotional states: comparison of the depression anxiety stress scales (DASS) with the Beck depression and anxiety inventories. Behav. Res. Ther. 33, 335–343. doi: 10.1016/0005-7967(94)00075-u, 7726811

[ref42] Luo A. He H. Mohamed S. Rosenheck R. (2018). Medical student attitudes towards people with mental illness in China: a qualitative study. Cult. Med. Psychiatry 42, 535–551. doi: 10.1007/s11013-018-9568-9, 29508204

[ref43] Mack S. Jacobi F. Beesdo-Baum K. Gerschler A. Strehle J. Höfler M. . (2015). Functional disability and quality of life decrements in mental disorders: results from the mental health module of the German health interview and examination survey for adults (DEGS1-MH). Eur. Psychiatry 30, 793–800. doi: 10.1016/j.eurpsy.2015.06.003, 26169476

[ref44] Mack S. Jacobi F. Gerschler A. Strehle J. Höfler M. Busch M. A. . (2014). Self-reported utilization of mental health services in the adult German population--evidence for unmet needs? Results of the DEGS1-mental health module (DEGS1-MH). Int. J. Methods Psychiatr. Res. 23, 289–303. doi: 10.1002/mpr.1438, 24687693 PMC6878535

[ref45] Mackenzie C. S. Knox V. J. Gekoski W. L. Macaulay H. L. (2004). An adaptation and extension of the attitudes toward seeking professional psychological help scale. J. Appl. Soc. Psychol. 34, 2410–2436. doi: 10.1111/j.1559-1816.2004.tb01984.x

[ref46] Ociskova M. Prasko J. Kamaradova D. Grambal A. Sigmundova Z. (2015). Individual correlates of self-stigma in patients with anxiety disorders with and without comorbidities. Neuropsychiatr. Dis. Treat. 11, 1767–1779. doi: 10.2147/NDT.S87737, 26229471 PMC4514318

[ref47] Ociskova M. Prasko J. Vrbova K. Kasalova P. Holubova M. Grambal A. . (2018). Self-stigma and treatment effectiveness in patients with anxiety disorders - a mediation analysis. Neuropsychiatr. Dis. Treat. 14, 383–392. doi: 10.2147/NDT.S152208, 29416340 PMC5790087

[ref48] Ostrom T. M. (1969). The relationship between the affective, behavioral, and cognitive components of attitude. J. Exp. Soc. Psychol. 5, 12–30. doi: 10.1016/0022-1031(69)90003-1, 42475469

[ref49] Perry L. R. Moorhouse T. P. Jacobsen K. Loveridge A. J. Macdonald D. W. (2022). More than a feeling: cognitive beliefs and positive—but not negative—affect predict overall attitudes toward predators. Conserv. Sci. Pract. 4:e584. doi: 10.1111/csp2.584

[ref50] Perte A. Albu M. (2011). Manual pentru Scalele de Depresie, Anxietate și Stres—DASS-21R. Cluj-Napoca: Editura ASCR.

[ref51] Rickwood D. J. Deane F. P. Wilson C. J. (2007). When and how do young people seek professional help for mental health problems? Med. J. Aust. 187, S35–S39. doi: 10.5694/j.1326-5377.2007.tb01334.x, 17908023

[ref52] Rosenberg M. J. Hovland C. I. McGuire W. J. Abelson R. P. Brehm J. W. (1960). Attitude Organization and Change: An Analysis of Consistency among Attitude Components. (Yales Studies in Attitude and Communication.). New Haven, CT: Yale University Press.

[ref53] Stuart H. (2006). Mental illness and employment discrimination. Curr. Opin. Psychiatry 19, 522–526. doi: 10.1097/01.yco.0000238482.27270.5d, 16874128

[ref9004] Thompson A. Hunt C. Issakidis C. (2004). Why wait? Reasons for delay and prompts to seek help for mental health problems in an Australian clinical sample. Social Psychiatry and Psychiatric Epidemiology, 39, 810–817. doi: 10.1007/s00127-004-0816-7 15669662

[ref54] Thornicroft G. Rose D. Kassam A. (2007). Discrimination in health care against people with mental illness. Int. Rev. Psychiatry 19, 113–122. doi: 10.1080/09540260701278937, 17464789

[ref55] Vlădescu C. Scîntee S. G. Olsavszky V. Hernández-Quevedo C. Sagan A. (2016). Romania: health system review. Health Syst. Transit. 18, 1–170, 27603897

[ref56] Vogel D. L. Wade N. G. Hackler A. H. (2007). Perceived public stigma and the willingness to seek counseling: the mediating roles of self-stigma and attitudes toward counseling. J. Couns. Psychol. 54, 40–50. doi: 10.1037/0022-0167.54.1.40

[ref57] Wang P. S. Lane M. Olfson M. Pincus H. A. Wells K. B. Kessler R. C. (2005). Twelve-month use of mental health services in the United States: results from the National Comorbidity Survey Replication. Arch. Gen. Psychiatry 62, 629–640. doi: 10.1001/archpsyc.62.6.629, 15939840

[ref58] Winkler P. Krupchanka D. Roberts T. Tulloch S. Murphy P. J. . (2017). A blind spot on the global mental health map: a scoping review of 25 years' development of mental health care for people with severe mental illnesses in central and eastern Europe. Lancet Psychiatry 4, 634–642. doi: 10.1016/S2215-0366(17)30135-9, 28495549

[ref59] Witte K. Thalmann M. Schulz E. (2024). *How should we Measure Exploration?*

[ref60] Wood A. L. Wahl O. F. (2006). Evaluating the effectiveness of a consumer-provided mental health recovery education presentation. Psychiatr. Rehabil. J. 30, 46–53. doi: 10.2975/30.2006.46.53, 16881245

[ref61] Yang J. Li Y. Gao R. Chen H. Yang Z. (2023). Relationship between mental health literacy and professional psychological help-seeking attitudes in China: a chain mediation model. BMC Psychiatry 23:956. doi: 10.1186/s12888-023-05458-5, 38129805 PMC10734200

[ref62] Zhao X. Lynch J. G. Jr. Chen Q. (2010). Reconsidering baron and Kenny: myths and truths about mediation analysis. J. Consum. Res. 37, 197–206. doi: 10.1086/651257

[ref63] Zhong S. L. Wang S. B. Ding K. R. Tan W. Y. Zhou L. (2024). Low mental health literacy is associated with depression and anxiety among adults: a population-based survey of 16,715 adults in China. BMC Public Health 24:2721. doi: 10.1186/s12889-024-20020-y, 39370527 PMC11456243

